# Genome scale transcriptome analysis of shoot organogenesis in *Populus*

**DOI:** 10.1186/1471-2229-9-132

**Published:** 2009-11-17

**Authors:** Yanghuan Bao, Palitha Dharmawardhana, Todd C Mockler, Steven H Strauss

**Affiliations:** 1 Department of Forest Ecosystems and Society, Oregon State University, Corvallis, Oregon 97331-5752, USA; 2 Department of Botany and Plant Pathology, Cordley Hall 2082, Oregon State University, Corvallis, Oregon 97331-2902, USA; 3 Center for Genome Research and Biocomputing, Oregon State University, Corvallis, Oregon 97331-7303, USA

## Abstract

**Background:**

Our aim is to improve knowledge of gene regulatory circuits important to dedifferentiation, redifferentiation, and adventitious meristem organization during *in vitro *regeneration of plants. Regeneration of transgenic cells remains a major obstacle to research and commercial deployment of most taxa of transgenic plants, and woody species are particularly recalcitrant. The model woody species *Populus*, due to its genome sequence and amenability to *in vitro *manipulation, is an excellent species for study in this area. The genes recognized may help to guide the development of new tools for improving the efficiency of plant regeneration and transformation.

**Results:**

We analyzed gene expression during poplar *in vitro *dedifferentiation and shoot regeneration using an Affymetrix array representing over 56,000 poplar transcripts. We focused on callus induction and shoot formation, thus we sampled RNAs from tissues: prior to callus induction, 3 days and 15 days after callus induction, and 3 days and 8 days after the start of shoot induction. We used a female hybrid white poplar clone (INRA 717-1 B4, ***Populus tremula × P. alba***) that is used widely as a model transgenic genotype. Approximately 15% of the monitored genes were significantly up-or down-regulated when controlling the false discovery rate (FDR) at 0.01; over 3,000 genes had a 5-fold or greater change in expression. We found a large initial change in expression after the beginning of hormone treatment (at the earliest stage of callus induction), and then a much smaller number of additional differentially expressed genes at subsequent regeneration stages. A total of 588 transcription factors that were distributed in 45 gene families were differentially regulated. Genes that showed strong differential expression included components of auxin and cytokinin signaling, selected cell division genes, and genes related to plastid development and photosynthesis. When compared with data on in vitro callogenesis in *Arabidopsis*, 25% (1,260) of up-regulated and 22% (748) of down-regulated genes were in common with the genes regulated in poplar during callus induction.

**Conclusion:**

The major regulatory events during plant cell organogenesis occur at early stages of dedifferentiation. The regulatory circuits reflect the combinational effects of transcriptional control and hormone signaling, and associated changes in light environment imposed during dedifferentiation.

## Background

*In vitro *regeneration is a common research tool and important method for plant propagation. It is also essential for most forms of genetic transformation, which require the regeneration of single transgenic cells into non-chimeric organisms [1,2]. Both embryogenic and organogenic regeneration pathways are widely employed, with the system of choice varying among species and research or propagation goal.

Organogenesis systems are more widely applied than embryogenic systems, particularly in dicotyledenous plants, because the explants and *in vitro *conditions are less complex and more robust. During organogenesis, explants are generally subjected to four sequential stages: direct or indirect callus induction, adventitious shoot (or root) formation, adventitious root (or shoot) formation, and micropropagation using axillary or apical meristem containing tissues based on either shoot or root cuttings.

About a half century ago, the developmental fates of *in vitro *explants were shown to be largely controlled by the balance of cytokinin and auxin [3]. When cytokinin is high relative to auxin, shoots are induced; when the reverse is true, roots are induced. When both hormones are present, but usually with dominance of auxin, undifferentiated growth of callus usually occurs. Although there has been a great deal of progress in identification of key genes that regulate embryogenesis and organogenesis [4-6], as well as genome scale studies of *in vitro *regeneration [7-9], the studies have focused on only a few species and specific regeneration systems.

Array studies of regeneration in *Arabidopsis *have focused on indirect regeneration via root explants rather than shoot explants [8], and used the Affymetrix ATH1 GeneChip which represents 22,810 genes. Root explants were pre-incubated on callus induction medium (CIM) for a few days, and then transferred to a cytokinin-rich shoot induction medium (SIM), an auxin-rich root induction medium (RIM), or fresh CIM, respectively. Nearly half (10,700 out of 22,810) of probe sets exhibited regulated expression profiles (FDR<0.01) across the time points of sampling. During early shoot development, 478 and 397 genes were specifically up-regulated and down-regulated, respectively. In rice, a monocot, somatic embryos regenerated from cell culture were used to induce shoots. By comparing gene expression at 7 days on SIM with somatic embryos using a 70-mer oligonucleotide microarray containing 37,000 probe sets, 433 and 397 genes were up-or down-regulated, respectively [9].

The genus *Populus *has emerged as a model system for plant and tree biology [10]. Its utility is likely to expand as a result of the publication of a complete genome of *Populus trichocarpa *(Torr. & Gray) produced by the USA Department of Energy Joint Genome Institute [11]. The value of poplar as a model tree results from its modest sized genome, facile transformation of selected genotypes, high capacity for *in vitro *propagation, rapid growth, extensive natural diversity, many natural and bred interspecific hybrids, and diverse environmental and economic values [12-14]. Its natural ability for vegetative regeneration, even from mature tissues, and its amenability to organogenic regeneration and transformation *in vitro*, has motivated a large number of studies of the biology and management of regeneration systems [1,2].

Microarrays have successfully identified many of the genes and regulatory factors related to specific physiological states in poplar. Wood formation has been intensively studied using microarrays. For example, changes in gene expression induced by gibberellic acid (GA) in the developing xylem was studied using a cDNA-based microarry analysis [15]. By comparing gene expression among stem micro-sections, the roles of many genes in xylem, phloem, and cambium development were characterized [16]. Subsequent to the completion of the poplar genome, two commercial oligonucleotide genome-scale microarrays were designed. One was produced by NimbleGen and another by Affymetrix. In *STM*-homolog over-expressing poplars, 102 and 173 genes were identified as up- or down-regulated by two-fold or greater, respectively, using a NimbleGen platform [17]. In a genome-wide expression analysis using the NimbleGen microarray platform, of *Auxin/Indole-3-Acetic Acid *(*Aux/IAA*) and *Auxin Response Factor *(*ARF*) in *Populus (Populus trichocarpa *clone Nisqually-1), the genes in a subgroup of *Aux/IAA *showed differential expression among different tissue types [18].

The goal of this study was to characterize the changes in gene expression that accompany dedifferentiation and organogenic regeneration in *Populus*, and compare them to results from *Arabidopsis *and other species. Characterization of the regulatory networks from poplar--with its distinct *in vitro *system and phylogeny compared to the other species studied to date--should give new insights into the conserved mechanisms for maintenance and regulation of plant stem cells. We conducted a genome-scale transcriptome analysis using the Affymetrix Poplar Genome GeneChip. It monitors more than 56,000 transcripts based on poplar genome and EST sequences. In this report, we describe the identities and biological roles of more than 9,000 unique regulated genes observed over five stages of regeneration.

## Results

We studied gene expression during dedifferentiation and regeneration of shoots via organogenesis. Similar in vitro methods are widely using in the regeneration and transformation of Populus and many other plant species. Two biological replicates were used for each of five time points from pre-induction to shoot regeneration, and the RNAs hybridized to genome-scale arrays.

### Callus and shoot development during regeneration

**Figure 1:**
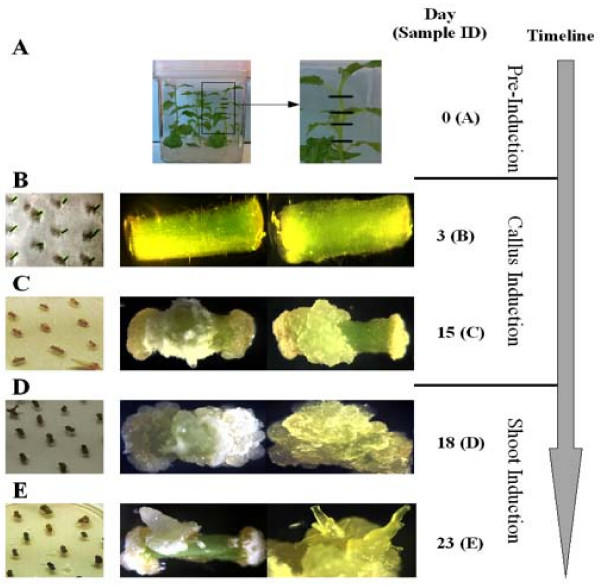
**Tissue sampled during *in vitro *shoot organogenesis**. Internode explants from *in vitro *micropropagation were sampled for RNA extraction at five sequential time points. They were first placed on callus induction medium (CIM) and then on shoot induction medium (SIM). The sample times were: (A) directly after removal from parent plants and prior to placement on CIM; (B) 3 days after placement on CIM; (C) 15 days on CIM; (D) 3 days on SIM after CIM treatment; and (E) 8 days on SIM after CIM treatment.

To determine the time points for taking tissue samples during *in vitro *shoot organogenesis, we carried out a preliminary regeneration experiment where 3 to 4 mm internodal stem segments (Figure 1A) were placed on auxin-rich CIM in dark for 15 days, then transferred them to cytokinin-rich SIM following our optimized transformation protocol (described under methods). No obvious morphological changes occurred during the first three days on CIM (Figure 1B). The explants began to form callus at the two ends starting at 7 days on CIM, and the size of callus continued to grow (Figure 1C and 1D). Individual or multiple shoot buds emerged from callus beginning from 8 days on SIM. Shoots were observed in approximately 10% of explants by 10 days on SIM (Figure 1E), and the percentage grew to around 20% at 20 days on SIM. Based on the above observations, explants were collected at 3 days both on CIM and SIM to detect early genetic regulation of callus induction and shoot induction, respectively. For the 8 day sample from SIM, only explants that had visible emerging shoots were chosen in an effort to ensure that transcriptional changes related to shoot regeneration could be detected.

### Quality assessment of array data

We inspected graphical images of the raw hybridization intensity for each of the 10 arrays, and found no severe spatial artifacts (See Additional File 1A) that would likely prevent accurate estimation of transcript expression levels over the 11 randomly located probes per transcript [19]. The Affymetrix quality report files (described under methods) -- which consist of average backgrounds, scaling factors, percentages of presence, internal controls, poly-A controls, and hybridization controls -- indicated that no significant flaws were detected (See Additional File 1B-G). Approximately 48,000 transcripts out of over 56,000 had detectable expression for at least one time point. The squared correlations between the two biological replicates ranged from 0.94 to 0.99 for each sample time (See Additional File 1H).

### Identification of differentially expressed genes

The number of differentially expressed genes identified by LIMMA (described under methods, see Additional File 2 for a list of the regulated genes at each stage identified by LIMMA) was 12,513, of which 9,033 had expression levels above those flagged as absent or marginal in the Affymetrix data quality reports at the stages when they are regulated. These 9,033 genes were considered in further analyses.

**Figure 2:**
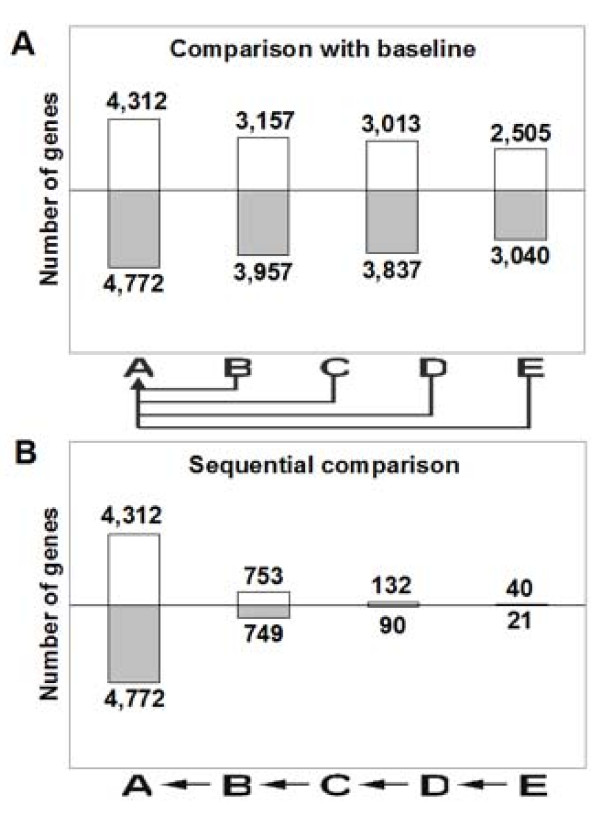
**Numbers of differentially expressed genes during regeneration**. (A) Differential expression calculated by comparison with the pre-induction stage (baseline). Numbers of differentially expressed genes were identified using Linear Models for Microarray Data (LIMMA). Empty bars above line are up-regulated genes; gray bars below are down-regulated genes. (B) Differential expression calculated by comparison with the prior sample point (sequential) using LIMMA as in A.

When expression at each stage was compared to that prior to regeneration (Figure 2A), we found up to 4,312 genes were up-regulated, and up to 4,772 genes were down-regulated. The largest number of regulated genes was identified at the earliest stage of callogenesis, though morphological changes were not yet visible at this time point. When comparing the expression at each time point with that of the previous time point, the difference among the numbers of differentially expressed genes declined nearly an order of magnitude with sequential time points (Figure 2B). In contrast to the thousands of regulated genes during early callogenesis, there were only 132 and 90 genes up- and down-regulated, respectively, during the early stages of shoot induction.

### Gene ontology categorization of differentially expressed genes

To identify the over-represented molecular functions and biological processes at each stage, we categorized the groups of the up-or down-regulated genes at each stage by their Gene Ontology (GO) class (See Additional File 3). Due to incompleteness of poplar GO annotations and the conservation of gene families between poplar and *Arabidopsis*, we used the *Arabidopsis *matches of the identified differentially expressed poplar gene as surrogates for GO categorization. We used normalized frequencies (described under methods) to test if a functional class was over-represented; when the normalized frequency of a functional class was larger than 1, this functional class was presumed to be over-represented in a group of genes.

Most of the GO biological process categories classes had similar numbers of genes that were up- and down-regulated (Table 1). However, at the onset of callogenesis--where the large majority of regulated genes were detected--there was a preponderance of up-regulated genes for the GO cellular components related to ribosome, cytosol, mitochondria, cell, wall, and endoplasmic reticulum functions. In contrast, there was strong down-regulation for chloroplast and plastid functions. For GO molecular function categories, a preponderance of up-regulation during the start of callogenesis was observed for structural molecule activity, nucleotide binding, and nucleic acid binding.

**Table 1:** GO categorization of differentially expressed poplar genes during in vitro organogenesis

		B vs. A		C vs. A		D vs. A		E vs. A	
GO category	Function category	Up	Down	Up	Down	Up	Down	Up	Down
Biological Process	response to stress	1.8	1.8	2.1	1.7	2.3	1.6	2.3	1.8
	cell organization and biogenesis	1.6	1.2	1.1	1.2	1	1.3	1	1.3
	response to abiotic or biotic stimulus	1.5	2	1.8	2	1.9	1.8	1.8	1.9
	developmental processes	1.5	1.3	1.4	1.4	1.3	1.6	1.5	1.6
	other metabolic processes	1.3	1.2	1.3	1.2	1.4	1.1	1.4	1.1
	other cellular processes	1.3	1.2	1.3	1.2	1.3	1.2	1.3	1.1
	protein metabolism	1.3	1	1	1	1	1	1	1
	electron transport or energy pathways	1.2	1.8	1.5	1.7	1.6	1	1.6	1.1
	transport	1.1	1.2	1.1	1.2	1.1	1.4	1.1	1.3
	DNA or RNA metabolism	1.1	0.4	0.6	0.5	0.6	0.6	0.6	0.6
	other biological processes	0.9	0.9	0.9	0.9	0.9	1	0.9	1
	signal transduction	0.9	1.4	1	1.4	1.1	1.6	0.9	1.8
	transcription	0.8	1.1	1	1.1	1	1.3	1	1.4

Cellular Component	ribosome	2.8	0.9	0.9	1	1.1	0.2	1.5	0.2
	cytosol	2.8	1.1	2.1	1.1	2.3	0.9	2.8	1
	mitochondria	2.6	0.9	1.9	0.8	1.9	0.8	1.8	0.7
	cell wall	2.2	0.9	2	1.2	2.1	1.3	2.5	1.3
	other cytoplasmic components	2	2.1	1.4	1.9	1.5	1.2	1.6	1
	ER	2	0.2	2.1	0.5	2.1	0.8	2.4	0.4
	other intracellular components	1.6	1.6	1	1.4	1	1.1	1.1	1
	Golgi apparatus	1.3	1.2	0.7	1.5	0.7	2	0.4	1.8
	nucleus	1.1	1.1	1.2	1.1	1	1.3	1	1.3
	plasma membrane	1.1	1.9	1.3	2.3	1.4	2.5	1.1	2.6
	other cellular components	1	1	0.9	0.9	0.9	0.9	0.9	0.9
	chloroplast	1	2.5	0.9	2.1	1	1.2	1.1	1.2
	other membranes	0.9	1.2	1.1	1.3	1.2	1.2	1.1	1.1
	plastid	0.9	4.9	0.9	3.9	1	1.4	1.1	1.2
	extracellular	0.8	0.6	1.1	0.8	1.2	0.7	1.4	0.5

Molecular Function	structural molecule activity	2.2	1.4	0.7	1.3	0.9	0.8	1.2	0.8
	other enzyme activity	1.8	1.3	2	1.3	2.2	1	2.2	1
	nucleotide binding	1.7	0.9	1.4	1.1	1.3	1.2	1.2	1.2
	nucleic acid binding	1.6	0.9	1.2	0.9	0.9	0.9	1	0.9
	transferase activity	1.5	1.2	1.9	1.2	1.8	1.3	1.9	1.3
	transporter activity	1.4	1.3	1.4	1.4	1.4	1.6	1.4	1.6
	hydrolase activity	1.4	1.1	1.3	1.2	1.2	1.1	1.4	1.1
	kinase activity	1.1	1.3	1.4	1.4	1.4	1.7	1.2	1.8
	DNA or RNA binding	1	1	0.9	0.9	0.9	1	0.8	1.1
	protein binding	1	1.3	1	1.3	0.9	1.5	0.8	1.6
	transcription factor activity	0.8	1.4	1	1.5	1.1	1.7	1.1	1.8
	other binding	0.8	1	0.8	0.9	0.8	0.9	0.7	0.9
	other molecular functions	0.7	0.8	0.7	0.8	0.7	0.8	0.6	0.8
	receptor binding or activity	0.5	0.7	0.7	0.6	0.6	0.6	0.6	0.7

The *Arabidopsis *homologs of the identified differentially expressed poplar gene were used for GO categorization. The percentage of each functional class in the poplar genome is assumed to equal to that in *Arabidopsis*.

### Clustering of differentially expressed genes

**Figure 3:**
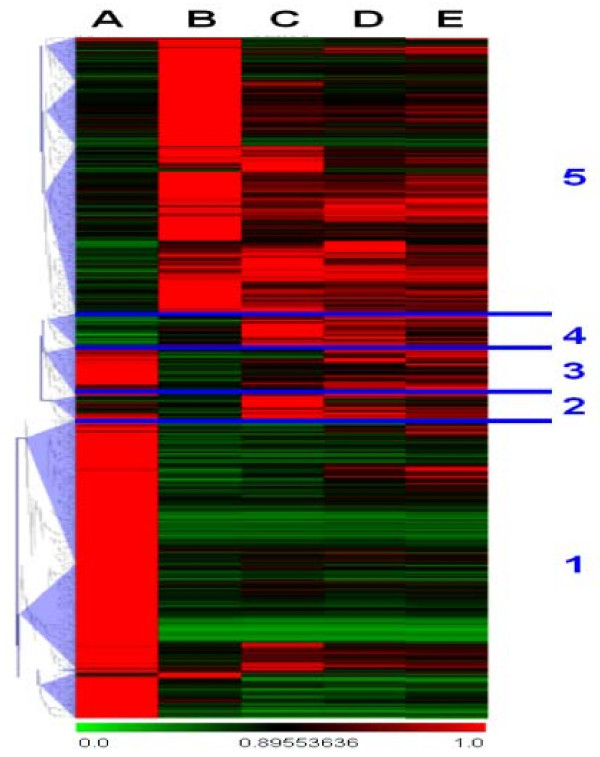
**Clustering of differentially expressed genes identified by LIMMA**. The ratios of gene expression at each time point and the highest level of expression of that gene among the five time points (i.e., a within-gene scale) were used for scaled clustering. Five distinctive expression patterns are labeled and discussed in the text (1-5). Expression scaling is indicated below clusters.

To identify genes with similar expression patterns during regeneration, we clustered the 9,033 genes identified by LIMMA that had expression levels above those flagged as absent or marginal in the Affymetrix data quality reports. At least five major clusters were visible (Figure 3). Prior to callus induction, about half of the regulated genes were strongly expressed, but most of these were shut down or repressed immediately and permanently upon callogenesis (Cluster 1, 5,434 genes). Only small numbers of genes formed the next three clades. One group included genes that were very weakly expressed prior to callogenesis, activated during late callogenesis, then sequentially shut down as shoot induction proceeded (Cluster 2, 587 genes). Another group's genes were strongly expressed then largely shut down throughout the rest of regeneration (Cluster 3, 1,028 genes); others were mostly turned off, further reduced in expression during initial callogenesis, then activated late in callogenesis and subsequently turned off during shoot induction (Cluster 4, 734 genes). Finally, a very large group of genes had very weak expression prior to regeneration, were activated rapidly and strongly during early callogenesis, then were largely down-regulated for the remainder of regeneration (cluster 5, 3,525 genes). There did not appear to be a cluster of genes that were specifically up-regulated during shoot induction.

### Clustering of differentially expressed transcriptional factors

We found that 588 transcriptional factors (23% of total) distributed in 42 families were differentially expressed (Table 2, see Additional File 4 for a list of the regulated transcription factors). Transcription factors involved in auxin signaling are among the most abundant regulated transcription factor families. Approximately 70% of Aux/IAA and 40% of ARF genes were up- or down-regulated during at least one stage. Other abundant families--involving at least 40% of its members--included the SRS, TLP, CCAAT-HAP2, GRF, and C2C2-Dof families.

**Table 2:** Regulated transcription factors during CIM and SIM

		**NO**.	**NO**.	B vs. A		C vs. A		D vs. A		E vs. A	
Gene family	Percentage	(regulated)	(total)	Up	Down	Up	Down	Up	Down	Up	Down
SRS	80.0%	8	10	3		5		2		4	
TLP	72.7%	8	11		6	4	4	2	6	2	4
AUX-IAA	69.7%	23	33	9	11	2	16		27		15
CCAAT-HAP2	45.5%	5	11		5		2		4		4
GRF	44.4%	4	9							4	
C2C2-Dof	42.9%	18	42	5	11		13	3	13		14
WRKY	38.5%	40	104	22	12	27	14	29	14	25	10
ARF	37.8%	14	37		13		10		13		10
HB	37.7%	40	106	15	23	7	22	7	24	6	24
AS2	36.8%	21	57	8	4	15	2	8	4	6	3
FHA	36.8%	7	19		7		8		4		4
ZIM	36.4%	8	22	8	3	7	2	5	3	4	2
GARP-G2-like	35.8%	24	67	2	19	4	14	6	19	3	15
TCP	35.3%	12	34		11	2	9		9	3	8
HMG	33.3%	4	12	2	2	2	2	2	3	2	3
LIM	33.3%	7	21		7		7		7		7
ULT	33.3%	1	3			1		1		1	
ZF-HD	32.0%	8	25		6		4		3	2	
E2F-DP	30.0%	3	10	2	2						
SBP	27.6%	8	29		8	4	5		7		6
CCAAT-HAP3	26.3%	5	19		5		3		3		3
Trihelix	25.5%	12	47	4	6	4	4	5	4	4	3
bHLH	25.0%	37	148	9	25	5	27	5	24	4	19
PLATZ	25.0%	5	20	5	4	4	2	3	2	3	2
bZIP	22.4%	19	85	4	22	2	18	4	17	3	17
Alfin	22.2%	2	9		2						
GRAS	20.8%	20	96	7	12	5	7	8	12	5	7
MYB-related	20.2%	17	84	5	14	3	6	2	7	2	6
HSF	19.4%	6	31	7		11		7		5	
MYB	19.0%	41	216	13	27	11	27	12	29	11	27
AP2-EREBP	18.9%	40	212	28	10	23	8	25	8	25	
NAC	18.6%	32	172	16	15	10	13	6	15	7	13
C2C2-CO-like	17.9%	7	39		10		6		7		5
C3H	17.9%	14	78	10	3	9	2	7	5	6	2
PcG	17.8%	8	45	2	6	2	3		3		2
C2H2	16.0%	13	81	5	11	3	9	3	9	2	9
C2C2-GATA	15.6%	5	32		2		2	3	3		
C2C2-YABBY	15.4%	2	13							3	
JUMONJI	15.0%	3	20					2			
TAZ	14.3%	1	7				1		1		
GARP-ARR-B	13.3%	2	15		4			2	3		3
ABI3-VP1	11.1%	12	108	9	4	9	2	10	2	9	6
MADS	10.8%	12	111		8	2	7	6	5	2	6
CCAAT-HAP5	10.5%	2	19					2		2	
PHD	9.3%	8	86	7	3	5		3	2	4	2

Total	22.8%	588	2576								

The JGI gene model IDs were downloaded from the Database of Poplar Transcription Factors (DPTF) and were searched against the list of the differentially expressed genes identified by LIMMA. The list is ranked by the percentage of the total number of each transcription factor class in poplar. The total number of regulated transcription factors were corrected for redundancy among the array probes; the number of up-or down-regulated transcription factors at each stage were not corrected for redundancy (i.e., multiple probe sets targeting the same transcript may be present).

**Figure 4:**
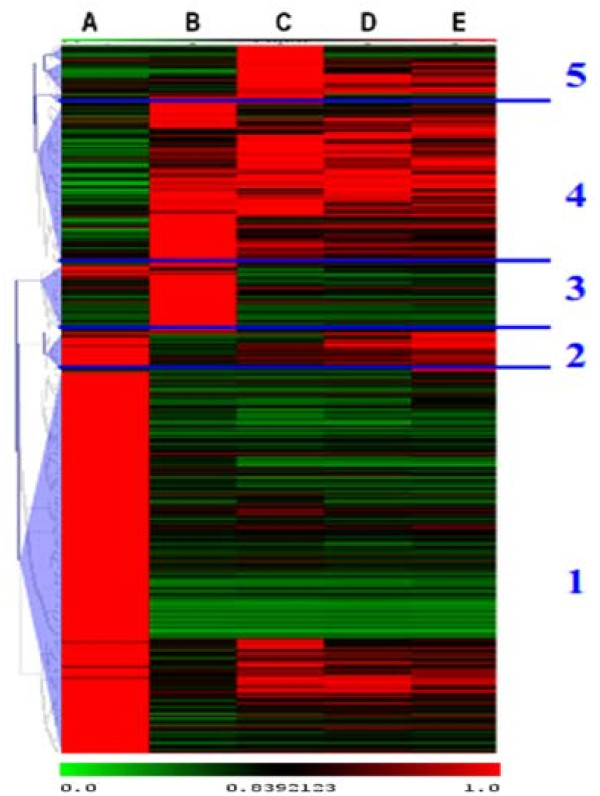
**Clustering of regulated transcription factors**. The ratios of the gene expression at each time point and the highest level of expression of that gene among the five time points (i.e., a within-gene scale) were used for scaled clustering. Five distinctive expression patterns are labeled and discussed in the text (1-5). Expression scaling is indicated below clusters.

When only transcription factors were considered in cluster analysis, several distinct clusters emerged, but were somewhat different in their patterns from the full gene list (Figure 4). Similar to the complete gene list, prior to callus induction more than half of the regulated genes were strongly expressed, but mostly shut down or repressed immediately and permanently upon callogenesis (Cluster 1, 316 genes). A small group of genes were also expressed prior to callogenesis and then shut down, but then were mostly reactivated during later stages of shoot induction (Cluster 2, 35 genes). Another small group of genes were largely unexpressed prior to callus induction, but then strongly up-regulated during early callogenesis and then largely deactivated again thereafter (Cluster 3, 52 genes). A large heterogeneous group had genes that were variably, but generally weakly, expressed prior to callus induction, but then reactivated at various times in callus and shoot induction (Cluster 4, 132 genes). Finally, a small group of genes were conspicuously and strongly expressed during late callogenesis, but weakly and variably expressed at other stages (cluster 5, 45 genes). As with the full gene set, there does not appear to be a cluster of genes that are specifically up-regulated during shoot induction.

### Auxin, cytokinin, and cell-cycle associated genes

**Figure 5:**
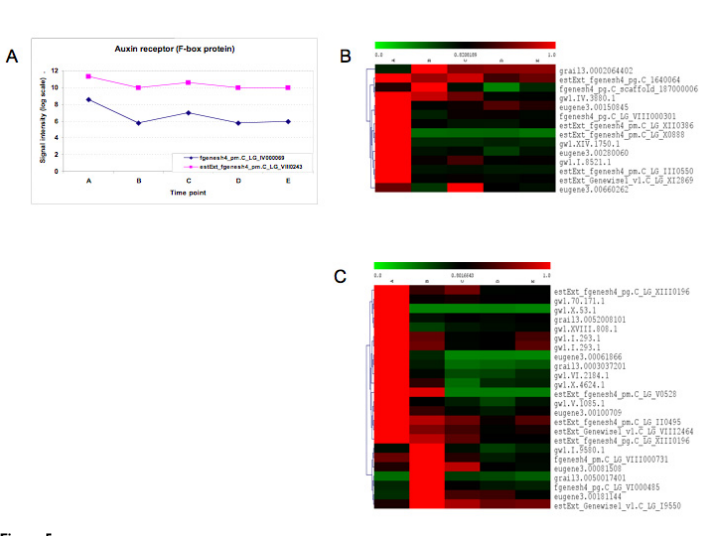
**Expression of regulated components in auxin signaling**. See Additional File 5 for a detailed list of the genes and their corresponding annotations. (A) The two regulated auxin-receptor F-box genes. (B) Clustering of regulated members of the ARF family. (C) Clustering of regulated members of the Aux/IAA family.

Two F-box genes were differentially regulated upon callus induction, and are closely related to *Arabidopsis TIR1 *(*Transport Inhibitor Response 1*) (Figure 5A, see Additional File 5 for a list of the regulated auxin signaling genes). After the early callus induction stage, their expression stabilized for the remainder of the regeneration period. A number of F-box genes are thought to take part in auxin signaling [20-22]. Twenty-three Aux/IAAs and fifteen ARFs were differentially expressed during at least one stage (Figure 5B and 5C). The majority of both classes of genes were down-regulated at the onset of callus induction and throughout subsequent regeneration, but specific groups of Aux/IAA genes were then up-regulated late in callus and during shoot development, or up-regulated during early callus induction and then down-regulated thereafter (Figure 5C).

**Figure 6:**
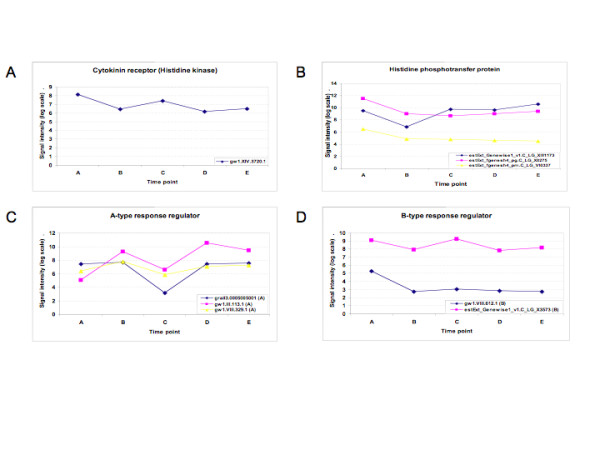
**Expression of regulated components in cytokinin signaling**. See Additional File 6 for a detailed list of the genes and their corresponding annotations. (A) A regulated cytokinin receptor (histidine kinase). (B) Regulated histidine phosphortransfer proteins. (C) Regulated A-type cytokinin response regulators. (D) Regulated B-type cytokinin response regulators.

A number of genes that take part in cytokinin signaling were regulated during regeneration (Figure 6, see Additional File 6 for a list of the regulated cytokining signaling genes). Key components of the cytokinin signaling and reception pathways include receptor kinases, phosphotransfer proteins, and various response regulators [23,24]. A putative cytokinin receptor histidine kinase gene was down-regulated upon callus induction. Three differentially expressed histidine phosphotransfer genes were down-regulated during callus induction, then up-regulated during subsequent growth and shoot regeneration. All three A-type response regulator genes were up- then down-regulated during callus development, then strongly up-regulated during shoot induction. Only one of two B-type response regulator genes was substantially down-regulated upon callus induction.

**Figure 7:**
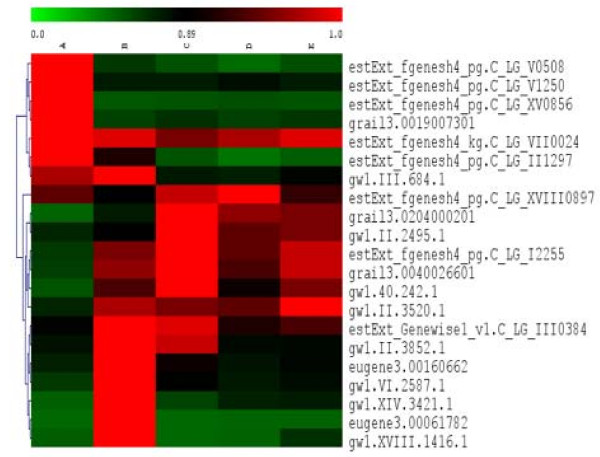
**Clustering of regulated cell cycle genes**. See Additional File 7 for a detailed list of the genes and their corresponding annotations.

Cell cycle genes are of obvious importance for regeneration, as slow growing explant tissues must be reactivated to grow rapidly during callus and shoot development. The cell cycle genes showed complex patterns of regulation, some being up- and others down-regulated at various points in regeneration (Figure 7, see Additional File 7 for a list of regulated cell cycle genes). A group was rapidly up-regulated, then mostly down-regulated after callus induction. Another group was not up-regulated until late in callus induction, but then was also mostly reduced in expression during shoot induction; some of these genes, however, did reactivate later in shoot induction. A third major group was strongly expressed prior to callus induction, then showed diverse patterns of reduced expression in subsequent stages.

### Comparison of regulated genes to Arabidopsis and rice

**Figure 8:**
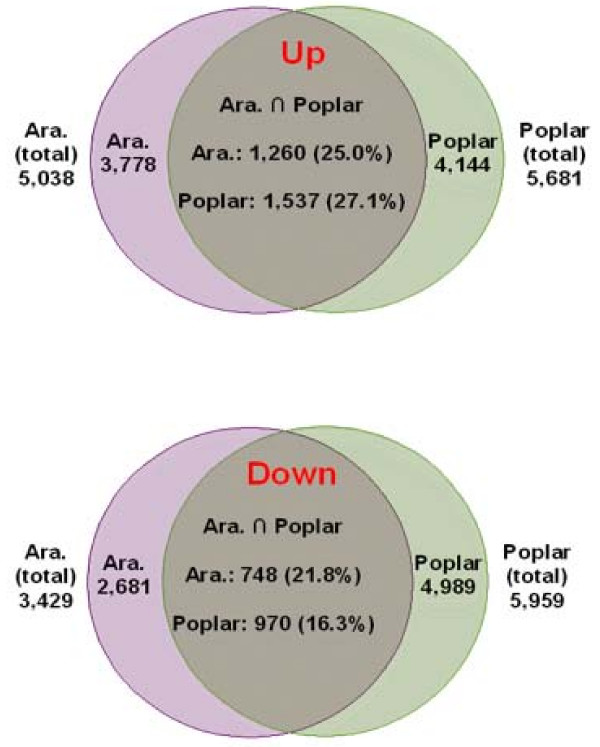
**Genes involved in callus induction common to Arabidopsis and poplar**.

**Figure 9:**
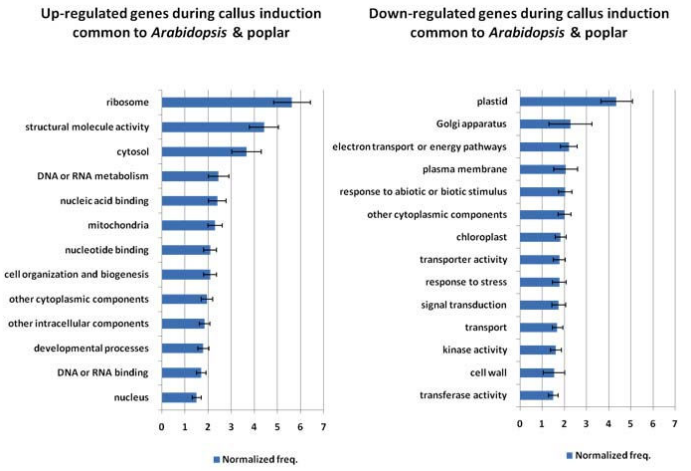
**Over-represented GO classes during callus induction common to Arabidopsis and poplar**. Bars are standard errors from bootstrapping of input datasets.

To identify genes whose function in regeneration is conserved among plant families, we compared our results to those of a similar microarray experiment in *Arabidopsis *[8] (See Additional Files 8, 9, 10, 11 for a list of the common genes). They reported changes in expression after four days on CIM to pre-induction root tissues, and found 5,038 up-regulated and 3,429 down-regulated genes at an FDR of 0.02. Our comparison revealed that 16% to 22% of down-regulated genes were in common, and 25 to 27% of up-regulated genes were in common, depending on the direction of comparison (poplar to *Arabidopsis*, or the reverse; Figure 8). Thus, approximately 2,000 genes were conserved in their basic roles among the two species. Of these genes approximately 8% were transcription factors. The largest GO classes of genes that were common and up-regulated include those related to cell growth, such as ribosome expression and DNA/RNA metabolism (Figure 9A). By far the largest common down-regulated class was genes related to plastid development (Figure 9B).

**Figure 10:**
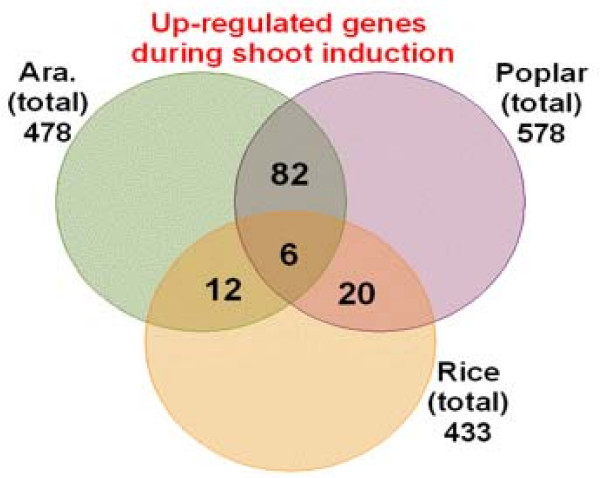
**Up-regulated genes during shoot induction common to *Arabidopsis*, poplar and rice**.

By using data on shoot regeneration from rice [9]*and Arabidopsis *[8], were able to compare up-regulated genes among all three species. Of approximately 500 genes from each species, only 6 were common among all three (Figure 10). There were more than 10-fold fewer genes in common between poplar and rice than there were between poplar and *Arabidopsis*. Among the 6 common genes, three are putative oxidoreductases with an NAD-binding domain.

## Discussion

Although some spatial variation in variability in hybridization intensity was visible on our arrays, we found that they gave a high degree of precision for estimates of gene expression. For example, 31,939 genes (out of a total 61,413 genes on the array) were flagged "present" for the both biological replicates prior to callus induction (i.e., above background, as determined by the Affymetrix software). Based on variance between biological replications after normalization and exclusion of any genes flagged "non-present" for one of the biological replicates, the mean, standard deviation, and coefficient of variation of signal intensity over biological replicates was 7.70, 0.20, and 3.18%, respectively. The mean standard error over biological replicates was 0.14 (1.84% relative to the mean). Thus, the precision in our estimates of approximately 2% is very small in relation to the large changes of gene expression observed, which often exceeded several hundred percent.

From the sequential comparisons of regulated genes, we found that there was a massive reorganization of gene expression shortly after the start of callus induction, but before visible changes in explant morphology were obvious. Changes in gene regulation after this point were far smaller, and decreased over time. Surprisingly, there were no substantial changes in gene expression observed after transfer to shoot induction medium. It may also reflect the observation that even after callus induction there was some meristematic activity observed in a number of explants, including the production of root initials. This may have coincided with a large and complex set of alterations in gene expression that are not substantially or simultaneously reset with the increase in cytokinin provided by the SIM medium.

The changes in GO categories reflect the large reorganization that tissues are undergoing during regeneration. Genes related to mitochondria, cell wall, ER, cell organization, and biogenesis were highly up-regulated during callus induction. This is a likely consequence of increased protein synthesis to support cell division and wall formation during callus induction. In contrast, chloroplast/plastid genes are strongly down-regulated gene during callus induction, which likely corresponds to the transition from autotrophy to heterotrophy at this developmental transition. It also likely reflects the suppressive effect of callus development in the dark in our regeneration system on light regulated, photosynthesis associated genes.

Two F-box proteins were regulated during regeneration. TIR1 and other three auxin F-box proteins have been suggested as auxin receptors involved in the regulation of auxin-responsive genes [20-22]. Auxin binds to TIR1 that is contained in SCF-like complex (SCFTIR1), which promotes the interaction between TIR1 and AUX/IAAs (reviewed by [25,26]. By comparison to auxin associated genes, only a small number of genes related to cytokinin signaling appear to be regulated in our dataset. However, the A-type response regulators and the pseudo-response regulator appear to be specifically induced during shoot induction, suggesting a direct role in cytokinin signaling. The type-A ARRs, are considered negative regulators of cytokinin signaling that are rapidly up-regulated in response to cytokinin [27].

There was strong and complex regulation of cell-cycle genes. In the JGI annotation, 110 genes have been assigned to GO:0007049, the cell cycle category [28]. Of these, 21 were differentially expressed during our regeneration treatments. Approximately half of these are hypothetical proteins, and 6 are cyclin genes. As expected given the rapid tissue growth that occurs during callogenesis, the majority (17 out of 21) were up-regulated around the time of callus induction. Among the four genes that were down-regulated during callus induction, estExt_fgenesh4_pg.C_LG_V0508 was identified as a cyclin dependent kinase inhibitor [29].

MYB proteins are a large group of transcription factors that have a wide variety of roles in development. For example, expression of many Myb genes is correlated with secondary wall formation, both in *Arabidopsis *and poplar [10,30]. During regeneration, we found that 41 (19% of the 216 poplar *MYBs*) showed regulated expression, and the number of down-regulated *MYBs *were roughly double the number of up-regulated *MYBs *at any stage. Not surprisingly, it appears that many Mybs play important roles in organogenesis.

The catalogs of regulated genes we have identified provide candidates for further analysis of their roles in *in vitro *development, and for modifying development for better control of regeneration. For example, many new gene family members and unknown genes could be characterized biochemically or via reverse genetic screens such as with RNAi or overexpression to identify their roles in control of regeneration. Induced expression of genes that appear to regulate cell cycle such as the cyclins, or of transcription factors that are associated with dedifferentiation such as some of the *MYBs*, might be useful for promoting regeneration of transgenic plants [31]. Microarray analysis of transgenic plants with these misexpressed genes would also provide insight into the regulatory networks in which they play a part. The modest level of conservation of the regulated gene sets between poplar and *Arabidopsis*, and the low level of conservation between poplar and rice, are not surprising given the considerably larger phylogenetic distance between rice, a monocot, and poplar and *Arabidopsis*, two dicots. Most important, however, is the likelihood that organogenesis in poplar was based on redifferention from shoot explants, whereas root explants were employed in *Arabidopsis; *and in rice, the distinctive embryogenic regeneration pathway was studied. These results suggest that transcriptome studies of a number of species and regeneration systems are needed in order to more fully understand--and thus to more rationally modify--the diverse *in vitro *regeneration pathways important to plant biology and biotechnology.

## Conclusion

The major transcriptional events in regulation of *in vitro *organogenesis in poplar occurred during the early stages of dedifferentiation. Nearly 10,000 genes were differentially expression during the onset of callus induction. A much smaller number of differentially expressed genes were detected at subsequent regeneration stages. A total of 588 transcription factors that were distributed in 45 gene families were differentially regulated. Genes involved in auxin signaling, cytokinin signaling, and secondary meristem regulation (eg. *MYB*s) were among the most abundantly regulated classes of transcription factors. Genes related to auxin signaling were highly regulated during regeneration. Two auxin F-box receptors, and more than a dozen Aux/IAAs and ARFs, showed differential expression. Differential expression of genes associated with cytokinin signaling included regulation of cytokinin histidine kinase receptors, two phosphotransfer proteins, and A-, B-type, and pseudo response regulators. Most of the identified cell cycle genes were up-regulated during callus induction. Substantial components of the regulatory circuits were conserved between *Arabidopsis *and poplar during callus induction, though different explants (stems vs. roots) were employed. Approximately one-fourth of the regulated genes in *Arabidopsis *were shared with poplar.

## Methods

### Plant material and culture conditions

Hybrid poplar clone INRA 717-1 B4 (female, *Populus tremula *× *P. alba*) was used for all experiments. Plants were propagated *in vitro *according to published protocols [32,33]. In brief, inter-nodal stem segments (3-4 mm in length) from *in vitro *micropropagated plants were cut and incubated on callus induction medium (CIM, MS containing 10 μM Naphthaleneacetic acid (NAA) (Sigma, St. Louis, MO) and 5 μM N6-(2-isopentenyl) adenine (Sigma) at 22°C in darkness for 15 days. Shoots were induced by culturing explants on shoot induction medium (SIM, MS containing 0.2 μM TDZ) (NOR-AM Chemical Co., Wilmington, DE).

RNAs were extracted separately from two batches (biological replications) that had been grown under the same growth conditions but three weeks apart in February 2007. For both, samples were collected at five time points: prior to callus induction, 3 days on CIM, 15 days on CIM (then transferred to SIM), and 3 days and 8 days on SIM. Approximately 10-15 stem explants from the same plate (~3 to 4 mm in length, with nodes removed) were pooled for RNA extraction for each biological replication

### Microarray platform

The Poplar Genome Array was designed by Affymetrix. It contains more than 61,000 probe sets representing over 56,000 transcripts and gene predictions. The probes are based on content from UniGene Build #6 (March 16, 2005), GenBank mRNAs, and ESTs for all *Populus *species (up to April 26, 2005) from the predicted gene set v1.1 from the *Populus *genome project (U.S. Department of Energy, Joint Genome Institute, http://genome.jgi-psf.org/Poptr1_1/[34]. The genome sequence is based on reads from a single tree of black cottonwood (*P. trichocarpa*) of the pacific northwestern USA [11].

### RNA extraction and quality examination

Total RNA was isolated and purified according to the RNeasy Mini Protocol for Isolation of Total RNA from Plant Cells and Tissues and Filamentous Fungi (QIAGEN Inc., Valencia, CA). A260/A280 ratios of RNA samples dissolved in 10 mm Tris pH 7.6 ranged from 1.9 to 2.1. The integrities of RNA samples were examined by the Agilent 2100 Bioanalyzer; their RINs (RNA Integrity Number) ranged from 8.6 to 10.0, and they showed no evidence of degradation.

### Array hybridization and quality assessment

The arrays were labeled and hybridized at the Center for Genomics and Biocomputing at Oregon State University [35] according to Affymetrix protocols. The quality of data was assessed by a series of parameters associated with assay and hybridization performance developed by Affymetrix. These include probe array image inspection, B2 oligo performance, average background, and noise values, poly-A controls (*lys*, *phe*, *thr*, *dap*), hybridization controls (*bioB*, *bioC*, *bioD*, and *cre*), internal control genes (3' to 5' ratios of *β-actin *and *GAPDH*), percent present, scaling, and normalization factors. The reliability and repeatability of this microarray platform was also evaluated by the correlations between the two biological replicates.

### Quantitative analysis

The probe-level data were normalized and summarized using the GC Robust Multichip Average (GCRMA) [36] algorithm using affylmGUI [37]. The algorithm computes gene expression summary values for Affymetrix data in three steps: a background adjustment using sequence information, quantile normalization, and finally expression value summarization. The summary values are based on a log2 scale.

Differentially expressed genes were identified by LIMMA (Linear Models for Microarray Data) [38,39]. LIMMA identifies differential expression via a modified t-test of gene expression between two treatments, using B statistics to rank differentially expressed genes. The BH P-value adjustment (Benjamini and Hochberg's FDR) was applied, using an adjusted P-value of 0.01 as a cutoff.

To reveal both global expression changes compared to the starting explant developmental state, and the specific expression changes taking place at each stage, two sets of contrasts between time points were used. First, the expression during each of the stages was compared with the baseline explant (CIM0). Second, the expression at each stage was compared with that of the previous time point.

Because of the well-established precision of the Affymetrix platform, and that our goal was to broadly cataloging patterns of gene expression, not to precisely estimate expression changes for specific genes, we did not conduct RT-PCR validation studies. Quality control studies have shown that results from RT-PCR are in agreement with microarray for genes with medium and high expression, and is not substantially more precise than the array platform employed itself [25].

### Biological interpretation

All annotation information for the Affymetrix Poplar Genome Array was retrieved from PopARRAY [40]. The annotation for each Affymetrix probe set ID consists of corresponding public ID, JGI poplar gene models, predicted *Arabidopsis *homolog, and functional annotation.

The JGI gene model IDs of all transcription factors were download from the Database of Poplar Transcription Factors (DPTF) [41]. DPTF catalogs known and predicted transcription factors from *Populus trichocarpa*. DPTF currently contains 2,576 putative transcription factors gene models, distributed in 64 families.

Hierarchical clustering was performed using MeV 4.0 (MultiExperiment Viewer) [42] with the Pearson correlation and average linkage model. The ratios of expression of a gene at each time point with its highest expression value among the five time points were used for scaled clustering.

GO annotation and categorization were done at the Bio-Array Resource for *Arabidopsis *Functional Genomics [43] with predicted *Arabidopsis *matches. The normalized frequencies were calculated as frequency of the class in the input data set divided by the frequency of the class in the whole genome. The class frequency was calculated as the ratio of the number of regulated genes in that class divided by the total number of genes in the class in the input data set, and the frequency of the class in the genome was calculated as the ratio of the total number of genes for that class in the genome divided by the total number of genes in the genome. The approximate reliability of over- or under- representation was evaluated by graphical presentation of standard errors based on 100 bootstraps of the input set. Because of the lack of a detailed genome annotation and associated statistics for poplar, the percentage of each functional class in the poplar genome was assumed to be approximately equal to that in *Arabidopsis*.

Comparative studies were carried out by comparing the regulated *Arabidopsis *homologs to a group of regulated poplar or rice genes detected under similar conditions. Data on *Arabidopsis *and rice was downloaded from the online supporting materials of the relevant publications [8,9]. For *Arabidopsis*, root explants had been preincubated on CIM for 4 days and then transferred to cytokinin-rich SIM. Among the monitored 22,810 transcripts on the Affymetrix ATH1 GeneChip, 5,038 (up-regulated) and 3,429 (down-regulated) genes exhibited regulated expression profiles with a false discovery rate of 0.01. During early shoot development, 478 and 397 genes were specifically up-regulated and down-regulated, respectively. For rice, somatic embryos generated from cell culture were used to induce shoots. By comparing gene expression 7 days on SIM with somatic embryos with a 70-mer long oligo microarray containing 37,000 probe sets, 433 and 397 gene were found up-or down-regulated, respectively (p-value < 0.05, ≥ two-fold change) [9]. For comparison of regulated genes identified between species, the *Arabidopsis *homolog ID (identification) numbers of the rice genes that were given in the online supporting tables [9], and the preferred *Arabidopsis *homolog IDs of the poplar genes from the PopArray database, were compared with the *Arabidopsis *IDs in [8,9]. A gene is considered to be in common with *Arabidopsis *if their *Arabidopsis *homolog ID matches the *Arabidopsis *ID.

## Authors' contributions

YB carried out the experiments, interpreted the data, and drafted the manuscript. PD and TCM gave extensive advice on microarray data analysis. SHS conceived of the study, provided funding, directed the overall project, and played a significant part in writing and interpretation. All authors read and approved the final manuscript.

## Supplementary Material

Additional file 1 Detail on quality assessment of microarray hybridization. Click here for file

Additional file 2 **Regulated genes at each stage identified by LIMMA.** 2a. CIM3-CIM0 down. 2b. CIM3-CIM0 up. 2c. SIM0-CIM0 down. 2d. SIM0-CIM0 up. 2e. SIM3-CIM0 down. 2f. SIM3-CIM0 up. 2g. SIM8-CIM0 down. 2h. SIM8-CIM0 up Click here for file

Additional file 3 Counts and percentages of regulated genes by GO category. Click here for file

Additional file 4 **Regulated transcription factors at each stage. **4a. CIM3-CIM0 down. 4b. CIM3-CIM0 up. 4c. SIM0-CIM0 down. 4d. SIM0-CIM0 up. 4e. SIM3-CIM0 down. 4f. SIM3-CIM0 up. 4g. SIM8-CIM0 down. 4h. SIM8-CIM0 up Click here for file

Additional file 5 Regulation of auxin signaling. Click here for file

Additional file 6 Regulation of cytokinin signaling. Click here for file

Additional file 7 Differentially expressed cell cycle genes. Click here for file

Additional file 8 Up-regulated genes at early callus induction common to Arabidopsis and poplar. Click here for file

Additional file 9 Down-regulated genes at early callus induction common to Arabidopsis and poplar. Click here for file

Additional file 10 Up-regulated genes during shoot induction common to Arabidopsis, poplar, and rice. Click here for file

Additional file 11 Down-regulated genes during shoot induction common to Arabidopsis, poplar and rice. Click here for file
