# Supplementary material for: Genome scale transcriptome analysis of shoot organogenesis in Populus
Source: BMC Plant Biol. 2009 Nov 17;9:132. doi: 10.1186/1471-2229-9-132 (PMC2784466; doi:10.1186/1471-2229-9-132)
Supplement: Additional file 1 — Detail on quality assessment of microarray hybridization. [file 1471-2229-9-132-S1.DOC]

**Supp1 Detail on quality assessment of microarray hybridization.**

*Quality measures are first described then results shown graphically for all measures below.*

(A) Artifact bias detected by residual image. The value at a probe is calculated as: signal intensity after normalization – signal intensity prior to normalization. Red: positive residue. Blue: negative residue.

(B) – (G) Background on quality parameters are provided in Affymetrix GeneChip Expression Analysis: Data Analysis Fundamentals (701021 Rev. 5, Page 36-40). For (B) – (H), A-E at X-axis indicates five time point for collecting samples. R1 and R2 indicate biological replicate group 1 and 2, respectively.

(B) Average background, typical ranging from 20 to 100.

(C) Scaling factor, usually around 3, less than 5 is considered acceptable;

(D) Percent of probes detected; 50% is common.

(E) Internal controls genes *β-actin* and *GAPDH* used to assess RNA sample and assay quality. Specifically, the ratio of the 3’ probe set to the 5’ probe set is generally no more than 3. However, a high 3’ to 5’ ratio of only one group of the internal control genes does not necessarily indicate RNA degradation.

(F) *Poly-A* controls used to monitor the entire target labeling process. All controls should be called “Present” with increasing signal value in the order of *lys*, *phe*, *thr*, and *dap*.

(G) Hybridization controls independent of RNA sample preparation, and used to evaluate sample hybridization; their signal values should reflect their relative concentrations (*bioB*:*bioC*:*bioD*:*cre* = 1.5:5:25:100).

(H) Correlation efficiency between biological replicates at five time points for collecting samples.

**A**


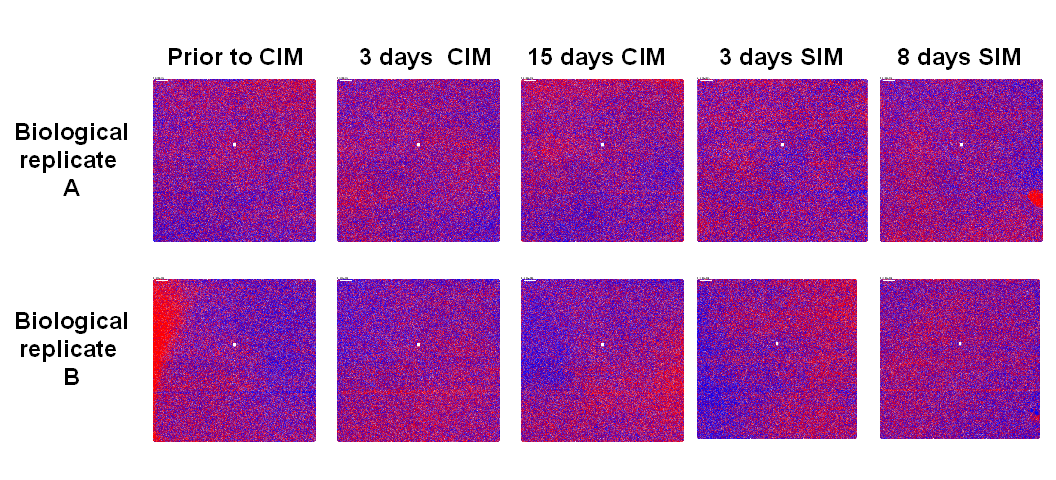


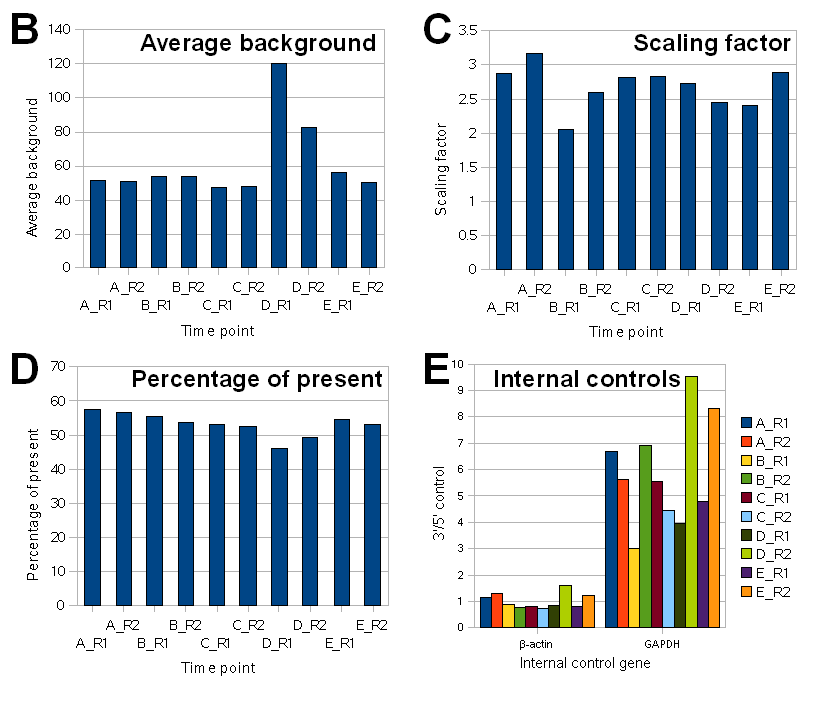


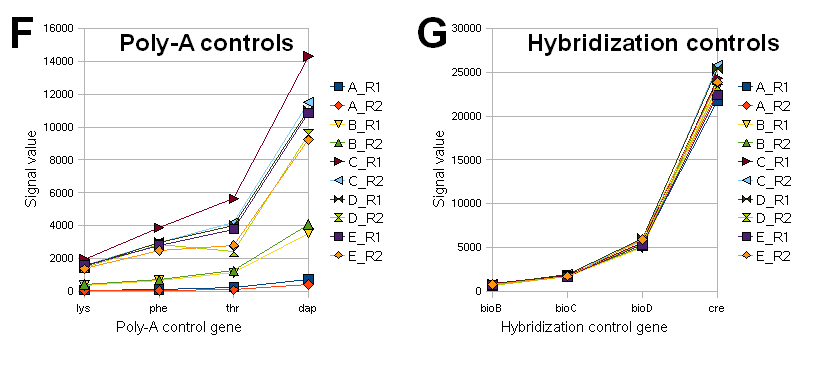


**H**

| **Time point** | **A** | **B** | **C** | **D** | **E** |
| --- | --- | --- | --- | --- | --- |
| **Correlation efficiency**  **between two biological replicates** | **0.987** | **0.990** | **0.979** | **0.940** | **0.974** |
